# Surgical Observations; Being a Quarterly Report of Cases in Surgery

**Published:** 1817-10

**Authors:** 


### *Surgical Observations; being a Quarterly Report of Cases in Surgery.* By CHARLES BELL. Part III. Octavo, pp. 124. Two Plates. London, 1817.

---

REPORT I. *Connexion between Pulmonary Diseases and Local Irritation, consequent on Wounds; and Surgical Osbervations.*

THIS is a curious subject, and deserves further investigation than it has yet met. Although the type of this symptomatic disease of the lungs does not differ materially from idiopathic inflammation, yet the circumstances are much changed, and the medical attendant is often put off his guard by the obscurity of the onset of the disease. Mr. Bell justly observes, that most men have from birth, what is not improperly termed a weak part; and when any constitutional disturbance takes place, this is pretty sure to suffer during the struggle. The lungs, in this variable climate, appear to be the organ predisposed, as it were, to disease; and the surgeon should keep this at all times in view. Mr. Bell exemplifies by alluding to the pulmonic disorder that so frequently follows the operation of fistula; but this we think is a different phænomenon from the common cases of pneumonia supervening on great surgical operations. We refer to Dr. Parry on this point. The instance of lithotomy is more to the purpose. Thus, after the extraction of a calculus, urine lodges in the track of the wound—irritation arises—bad matter is formed in the pelvis, and by the side of the rectum—and about the third week, pulmonic inflammation steals on the patient, and snaps the thread of life. Every one then regrets that such an untoward complaint should have supervened; but no one suspects that the operation had any thing to do with it!

Mr. B. thinks that it is frequently through *constitutional irritation* that the lungs become affected in cancerous mammæ. But it is on the severer operations, and especially on compound fractures, that pulmonitis more frequently supervenes. This is exemplified by several interesting cases, of which we shall only notice one.

"A coal-heaver was thrown between the body of his waggon and the wheel when in motion. The tibia had been rickety and curved, so that two parts of the arch of the bone were broken, and the integuments torn off the face of the bone. There was at first considerable bleeding from betwixt the ends of the fractured bones, and from the saphena vein. I was of opinion that this man's leg should be saved. He died on the morning of the 7th. There was no tumefaction, or erysipelatous sloughing of the skin; but he had delicate lungs, and was suffering from a chronic attack of inflammation and cough at the time of the accident."

An instance is related of disease excited in the lungs by the irritation of an old gun-shot fracture. The gentleman had received a musket shot in the pelvis, which could not be found. This was at the battle of Thoulouse. Mr. Bell long afterwards discovered the ball lodged in the thigh bone. The irritation of the wound was keeping up inflammatory action; and three months after this, the gentleman came to town, "hectic and sinking in consumption, with difficulty of breathing, a short cough, and purulent expectoration."—This we do not think a good example of the connexion between pulmonic inflammation and wounds; but rather a common instance of hectic fever from local irritation, the same as would have taken place in a lumbar abscess.

Disease of the lungs also follows amputation, of which there are two examples in the Middlesex Hospital. One is a coachman, who, having suffered amputation after a compound fracture of the leg, is spitting matter, and has had hæmorrhage from the lungs. He is considered to be lost. The other is a young man who has suffered high amputation of the arm, and has just recovered from an acute attack of inflammation in his chest.

"On reviewing this subject, it will be apparent, that injuries to the frame, whether the effect of wounds or of surgical operations, by exciting a high state of irritation, tend to disorder the lungs; and that especially if there be any tendency to disease in this organ, however latent before the injury, it will be developed; and increasing the constitutional disturbance, endanger the patient's life. It also appears, that as wounds, by their sudden and more violent inflammation, produce a corresponding acute attack on the lungs, so do they often, by a more gradual influence, bring on a phthisis.

"When a man has received a severe wound, and the inflammation changes to a dark colour, with gleety discharge, and he becomes restless and irritable, with a quick pulse; if, when in this state, he be seized with a pain in his side, he is in extreme danger: and if this attack be not guarded against, he will have difficulty of respiration; he will not dare to push his voice; we shall find that he speaks quick, and in a whisper; he pants like an over driven sheep; the muscles of his chest and neck are in continual exertion, and his nostrils are expanded and his lips tremble.

"If his weak and feverish state conceal this inflammatory attack, or render the practice necessarily feeble; if, with this inflammation in the lungs, the previous exhaustion of the patient, or the influence of the hospital, have reduced the powers of the body, he will die; and on dissection, the lungs will exhibit the marks of recent inflammation—coagulable lymph exuded, purulency on the surface of the lungs or in the bronchia, and congestion or abscess in their substance. How often are we inclined to say, that the patient who dies after a great operation, has fallen a victim to abscess in the lungs, without duly considering how much the stimulus of the knife has to do in exciting this mischief? A knowledge of this connexion betwixt wounds and the state of respiration, will make us careful to defer all operations when any tendency to disease prevails: and if this be not possible, it will make us watchful of the first symptoms, and active in our practice.

"Whilst the dissections prove the necessity of the most active remedies to subdue the inflammation of the lungs; the consideration of how much depends on the wound, will make us desist from all irritating applications at such a juncture, and seek to soothe and relieve the local inflammation.

"In these observations I have sought to establish my facts by the surer method of anatomy; which, on re-perusing the cases, I find gives a very fatal character to this inflammatory attack on the chest. Yet I will not introduce examples of the cure of such attacks, to vindicate our practice at the hazard of weakening the impression which I hope I have made on my readers." P. 259.

Report II. *On the Ligature, and on Wounds of Arteries.*

Here Mr. Bell makes a desperate attack on the "ingenious trifles" that have "been offered as improvements," in operating and securing arteries. He reprobates the new mode of tying with small ligatures, and drawing them so tightly as to cut the inner coats of the vessels.

"I confess (says he) that I could not have imagined to what an absurd length the opinion would be carried by practical surgeons, *as if in emulation of the experiments of young men preparing for their Theses.*" P. 260.

We need not inform our readers who are the dead and who are the living writers that are here aimed at. The living can defend themselves; but the genius of Jones ought to have sanctified his fame and his merits, which are here attempted to be blasted by Mr. Charles Bell. We would, however, as friends, recommend him to be cautious of the language in which he indulges; he is probably preparing for himself much trouble and much mortification. The late experiments of Crampton and others are thrown into ridicule by the following tests.

"To correct this erroneous, because partial view of the subject,

I made the following simple experiment:—I put a cord around the carotid of a dog, without drawing it. The ligature lay in contact with the coats of the artery, but did not press upon them, nor interrupt the flow of blood through them. I was certain of the result; a clot formed where the artery was irritated by the presence of the ligature, and the vessel was obstructed at that part." P. 261.

Into this dispute respecting the size and tightness of ligatures, we shall not enter, as competent judges and men of great experience, are now employed in ascertaining the truth and merits of both sides of the question.

The following very interesting case of wound of the brachial artery, we shall condense for our readers' information:—A shoe-maker was wounded on the 15th of September last by a pointed knife, which entered on the fore and outer side of the biceps muscle. He was brought into the hospital after losing nearly a pail-full of blood. The wound was enlarged, and various means were ineffectually used to cause the artery to bleed. He was put to bed, with the tourniquet slackly on. In half an hour, a jet of arterial blood came away by coughing. The wound was again enlarged—the coagulum scooped out, and the cavity sponged, which was now large, dark, and ragged. The artery could not be found—the tourniquet was loosened, but no blood flowed. At length the median nerve being raised, the artery appeared beneath, and being found wounded, was securely tied above and below the orifice in its calibre. The wound was brought together and closed. Next day all was doing well, and Mr. Bell thought the patient "quite safe."

18th. A brownish redness on the elbow, and matter is already formed, and can be pressed from the lower part of the fore-arm. Pulse cannot be felt at the wrist.

19th. Pulse intermittent; back of the arm is œdematous, and of a yellow colour. Two spots of mortification on the inside of the arm. Tepid fomentations with spirits; poultices.

20th. Omnia exacerbata. Features sunk; pulse quick, small, and intermittent. Arm more swollen towards the shoulder.—*Evening*. Highly delirious. After various turns for better and worse, he finally sunk on the 13th of October: a month from the receipt of the injury.

"On dissecting the brachial artery, the places where the ligature had been applied, were not distinguishable but for the stopping of the wax injection; singular as it may appear, I should not have imagined the continuity of vessel could have been destroyed, had I not seen the trunk tied, and seen the ligatures come away; had I not, I may say, seen the artery thus cut through at two places." P. 277.

*On Hæmorrhage from the lower Orifice of Arteries.*

A Frenchman had his thigh bone shattered with a grape-shot, and amputation was necessary. The artery was compressed on the groin, and then the vessel was cut down upon by Mr. Bell, and tied. On making a division of the artery below this, blood flowed in distinct jets from the lower orifice, shewing the freedom of inosculation, and the necessity of tying both ends of a wounded artery.

Another case is related, where the humeral artery was wounded, and only the upper mouth tied. The patient bled to death by successive hæmorrhages from the lower orifice.

Case 3d.—*Communicated by a Student.* A man was wounded in the groin, and the inguinal artery was opened near the edge of Poupart's ligament. After great hæmorrhage, and ineffectual attempts to catch the artery, it was necessary to cut down upon and tie the external iliac. A hæmorrhage of black blood took place on the tenth day, of which the patient expired. The femoral vein was found wounded; a coagulum in the upper, but none in the lower orifice of the artery. The medical men present thought the blood came from the femoral vein—Charles Bell thinks it came from the artery.—*Non nostrum inter illos tantas componere lites.*

Mr. Bell here suggests the propriety of making an incision above a wounded artery, so as to compress it while it is taking up. Thus—

" — if a man be wounded in the axilla, and the sponge suppresses the bleeding, but leaves no room for tying the axillary artery, I have recommended that a small incision be made above the clavicle, to let the finger down upon the first rib, where it will effectually compress the artery, and let the bleeding vessel be securely tied. P. 287.

We have so often seen the axillary artery divided in shoulder-joint operations, and the hæmorrhage restrained effectually by simple compression above the clavicle, that we cannot but think the above proposal useless, if not dangerous.

Here Mr. Bell alludes to the two operations performed on the carotid artery in this country, where the great branches of the external carotid had been wounded. In his usual sarcastic style, he remarks,

"I think a *pupil* of Windmill Street would have proceeded differently when he made the incision on the neck. *He* would have compressed the carotid betwixt his fore-finger and thumb, and using them as a tourniquet, he would thus have arrested the blood, and have had an opportunity of seeing the bleeding vessel; and he would have tied the external carotid, not the common carotid." P. 288.

Notwithstanding the blaze of light which illuminates the Windmill Street Theatre, we would prefer trusting our necks in the hands of an Abernethy, to those of even the *pupil* of a Charles Bell.

### *On the Question of Amputation.*

The following case exhibits a memorable example of the evil consequences of tight bandaging in cases of fracture.

"An elderly man, a lean artificer, fell from a height and broke both the bones of the fore-arm, the surgeon had put on splints and a bandage, with due attention to the inevitable degree of swelling; which must accompany so great an injury. In the morning, when brought to the hospital and examined, the hand was swollen, dark, and cold: and although the bandage was immediately undone, the impression was made, and vesications appeared upon the surface. In a few days the whole fore-arm and hand mortified.

*Consultation.* "The arm is acknowledged to be irrecoverably gone; a line of separation is commenced in the blush on the skin which goes round the elbow, but the age and circumstances of the patient forbid the hope of the fore-arm being cast off at the elbow joint. The mass of mortification is at this moment weighing upon the constitution. The consultants agreed that it could not be expected that the mortified arm should slough off, and that an operation afforded the only hope.

"Then the question arose, was this the proper time to amputate? It was observed, on the one hand, that the operation should not be deferred: 1. Because the line of separation was commenced, shewing the system capable of an effort. 2. Because the man's pulse was better through the influence of wine, bark, opium, and ammonia; an effect which could not be relied on for any considerable time. 3. The skin was free above the line of mortification and inflammation, which might not be the case tomorrow. Against this it was suggested, that the man was better to-day than he was yesterday, and so might be in a situation better able to undergo the operation to-morrow."

14th. "This man's tongue is cleaner, his pulse is firmer, his senses are improved, he has had less delirium; he is more alive to what is going on around him. But a hardness of the skin, with inflammation, is spreading up the arm on the inside; advice was again asked, and amputation performed."

*One o'clock.* "The patient bore the operation well; indeed it was quickly done, so that I hoped there was not much shock to the constitution. He lost no arterial blood. It was observed, that the smaller vessels were more active than the trunk. When they were tied, the profunda humeri was seen to pulsate; but the humeral artery, though tied, distinct and separate from the surrounding substance, did not pulsate. This was a singular circumstance, and is to be explained by the consideration that the main artery belonged to the fore-arm which was dead, and the branches to the arm where there was still circulation."

*Five o'clock.* "He has taken some generous soup, and a little pudding; every four hours he is ordered four spoonfuls of Port wine, in which the extract of bark has been infused, and he is to have an opiate in the evening."

*Evening.* "His pulse rose from the operation, and remains fuller than in the morning; he is quite sensible, and vain of his non-chalance."

*Second day.* "He slept six hours last night. Countenance better; tongue cleaner; pulse much fallen and quicker.—*Third day.* Pulse 92 and stronger; the dressings have been partially removed from the shoulder; the integuments are not so full as I could wish. The wine is taken from him; he is indulged in ale, and it agrees with him.—*Fifth day.* He was dressed to-day; the stump discharges a considerable quantity of thin pus; pulse 96, and tolerably steady.—*Sixth day.* A hardness of the integuments has taken place, yet the pus is not bad. The pulse numbers as yesterday, but there is more thrill in it; and the tongue is brown. He is, if possible, to take more nourishment, and his wine is to be resumed.

"This man continued to sink; rallied again, under the most diligent attention of the apothecary and house surgeon, who rose, during the night, to feed him and give him his brandy in his gruel; but the stump dried up, and he died." p. 297.

The following note, made at Brussels after the battle of Waterloo, must be taken notice of by some of the surgeons employed there, or else they are void of feeling!

"To-day I have examined one division of the hospital. These cases are very peculiar, and there is a sameness in their character in which I cannot be mistaken. You see a small hole, such as might admit a bougie, the thigh or leg enormously swollen, and from the position you see that the bone is broken. I push my finger slowly into this hole; it enters by a sort of stricture, but all within is a vast cavity, and the jagged bones meet the finger. As the finger is withdrawn, there gushes out a stream of thin bloody matter. Such is the state of the gun-shot fracture fourteen days after the infliction of the wound, when nothing has been done to afford relief. I have, on other occasions, in different words, given the same description.—You see here the great swelling and inflammation of the limbs; you wonder to see the wound so small; but there is something which you cannot see: the range which the finger has when introduced into the wound of the thigh, the putrid matter, with resolved blood, which spouts out, and the bones, sharp as pins, which are discovered within." p. 319.

A little farther on, he says,

"In several of these, the stump was left *without even the loose pieces of bone being picked off the face of the wound.*" p. 319.

At page 321, Mr. Bell states, that—

"— the extremities of the bone are not subject to necrosis, and, therefore, it can never be necessary, on this account, to dissect the head of the humerus or femur from their articulations."

Mr. Bell gives an animated sketch of the scenes which passed in the military hospitals at Brussels after the battle of Waterloo. At the hospital of the Gendarmerie, he amputated, he thinks, about twelve times,* and he complains that his exertions there have called forth no acknowledgement, and that all his applications for a return of the men operated on have been disregarded.

The question of early amputation is here again brought forward, and left undecided in a long string of queries.

### *Report on the Nitro-Muriatic Acid Bath.*

When a patient presents himself greatly reduced by suffering and despondency, loathsome to himself and his friends, from the sequelæ of syphilis, and half poisoned by mercury, how valuable must that remedy be, which, without further weakening the vital powers, can clear the skin of ulcers, restore colour and animation to the countenance, and thus enable us to return the patient to society. When such are the effects we witness, of what real consequence is any difference of opinion concerning the action of the medicine, or the nature of the disease? The cases which Mr. Bell has laid before us, will show, that the picture is not overcharged. They are all hospital cases, drawn up at the bed-side of the patients. Mr. Bell wishes it to be understood, that he is not offering a substitute for mercury in syphilis, but only a mode of removing a very disagreeable train of symptoms, in such cases only as were rendered obscure by mismanagement, or in which mercury had been inefficient or hurtful. Without denying the existence of new diseases, Mr. Bell

* A staff surgeon, who was present, has informed us, that the number was considerably greater. Rev.

believes, that the subject of pseudo-syphilis has acquired importance from the multitude of cases of syphilis improperly treated, which offer themselves in public institutions.

"We find (says he) practitioners screening themselves under the authority of great names, and believing themselves to be blameless, because dealing with some new form of disease, when in fact they have mistaken a common case of syphilis. By local applications, the hardness of the primary sore is kept up, while the mercurial course is pushed with a design to destroy the hardness; — then comes mercurial sore throat, and they have entered the labyrinth!"

Syphilis often shows itself weakened, almost worn out, but not extinguished, liable to break out in circumstances favourable to its developement, but still in a state to be subdued by the lesser remedies. Hence it is that we see so many patients covered with sores and cutaneous eruptions, whose constitutions have been destroyed by repeated long courses of mercury.

CASE 1st. — *John Basham.* This patient had a discharge, with phymosis, nine months before he was admitted, with no sore on the penis at any period of his illness. He injected different lotions between the glans and prepuce, taking three mercurial pills every day for three or four months. This course of mercury failed in producing soreness of the gums. Before he had ceased taking the pills, eruptions appeared on the face and different parts of the body, followed by sores and pains of the bones. At his admission, he had a sore on the calf, with sharp edges, which appeared as if bit out. The whole of the left cheek, part of the right, and forehead, covered with a dry eruption. The end of the nose disfigured, and a great part of the upper lip eaten away. Besides sloughy sores, and scars on several parts of the body, he complains of pain over the ulna, increased when in bed. — Sept. 25. After using the nitro-muriatic bath, morning and evening, for a week, with a pint of dec. sarsæ, there appeared an evident amendment. A dropsical swelling appeared, which was attributed to the previous loss of blood he had sustained. He had lost a great deal of blood from the ulcer on his lip having opened the labial artery. The relief he felt from this, induced his surgeon to pursue his cure by bleeding him eleven or twelve times, which stopped the discharge of the ulcers. — Nov. 11. The bath continued for twenty minutes, morning and evening. The forehead and face are clear of all foulness; the ulcer of the leg has filled up.—Nov. 27. This man was dismissed entirely well, the sores being healed, and the dropsical swelling having disappeared without his gums being affected.

*Case of Joseph Bray.* He had used the mercurial ointment for a month, for a sore on the penis and a buboe. The sore was healed and the buboe was better; but three months after, having taken cold, the axillary glands inflamed, the open buboe began to enlarge, and his throat became very sore. Having been tampered with by a quack doctor in the Minories a reasonable time, he came to the hospital July 8th, 1816. He continued the mercurial course in the hospital three months, but on the whole was considerably worse than when he entered; the sores continuing to spread, though his throat was well. He then was put on the use of the nitro-muriatic acid bath, which he continued with increasing advantage for a month. The sores in the groin healed first, and the abscess in the axilla was evidently kept open only by the motion of the part. The sores were dressed simply, and the bath omitted: in a month after, he left the hospital perfectly cured, and with a healthy complexion.

It will be noticed, says Mr. Bell, that in all those cases where the bath proved so advantageous, a great deal of mercury had been previously used. Mr. B. prescribed the bath to several out-patients with excellent effect. They had been long on his list, and he was heartily weary of them.

"One young man had ulcers and sinuses on his thigh, who went through all the usual course of medicine; such as the decoct. sarsæ, alterative doses of the blue pill, Plummer's pill with bitter infusion, the corrosive sublimate in solution, decoction of bark with soda; besides dressings of various kinds, and compresses and bandages. He returned to me, month after month, without amendment; but I found that three weeks use of the bath to his feet, and washing his sores with the water of the bath, made him entirely well."

Mr. Bell has observed obvious constitutional effects arising from the bath. It produces bilious stools, oppression, and head-ache; in two instances ptyalism. Uniformly, after its use, the patients have become more plump, and confessed themselves in better health than usual.

Some observations follow from the pen of Dr. Scott; but as we have reviewed his paper, in the Journal of Science, on the same subject, we need not repeat the observations here.

Upon the whole, although this Report is somewhat inferior to the preceding, yet it is far from being devoid of interest, and we still wish Mr. Bell success. He may not thank us for our advice; yet we think there is something in his manner much calculated to create him enemies. His cavilling with the army surgeons does him no good, and may occasion him much mischief.

P. S. We can inform Mr. Bell, that not one of his operations succeeded at Brussels. If he doubts this statement, and insists on our authority, we will give it. It is authority which cannot be controverted.

